# Prediction of manifest refraction using machine learning ensemble models on wavefront aberrometry data

**DOI:** 10.1016/j.optom.2022.03.001

**Published:** 2022-04-14

**Authors:** Carlos S. Hernández, Andrea Gil, Ignacio Casares, Jesús Poderoso, Alec Wehse, Shivang R. Dave, Daryl Lim, Manuel Sánchez-Montañés, Eduardo Lage

**Affiliations:** a Department of Electronics and Communications Technology, Escuela Politécnica Superior, Universidad Autónoma de Madrid, Spain; b PlenOptika, Inc., Boston, MA, USA; c Instituto de Investigación Sanitaria Fundación Jiménez Diaz, Madrid, Spain; d Department of Computer Science. Escuela Politécnica Superior, Universidad Autónoma de Madrid, Spain

**Keywords:** Machine learning, Subjective refraction, Wavefront aberrometry, Portable autorefractor

## Abstract

**Purpose:**

To assess the performance of machine learning (ML) ensemble models for predicting patient subjective refraction (SR) using demographic factors, wavefront aberrometry data, and measurement quality related metrics taken with a low-cost portable autorefractor.

**Methods:**

Four ensemble models were evaluated for predicting individual power vectors (M, $J_{0}$, and $J_{45}$) corresponding to the eyeglass prescription of each patient. Those models were random forest regressor (RF), gradient boosting regressor (GB), extreme gradient boosting regressor (XGB), and a custom assembly model (ASB) that averages the first three models. Algorithms were trained on a dataset of 1244 samples and the predictive power was evaluated with 518 unseen samples. Variables used for the prediction were age, gender, Zernike coefficients up to 5th order, and pupil related metrics provided by the autorefractor. Agreement with SR was measured using Bland-Altman analysis, overall prediction error, and percentage of agreement between the ML predictions and subjective refractions for different thresholds (0.25 D, 0.5 D).

**Results:**

All models considerably outperformed the predictions from the autorefractor, while ASB obtained the best results. The accuracy of the predictions for each individual power vector component was substantially improved resulting in *a* ± 0.63 D, ±0.14D, and ±0.08 D reduction in the 95% limits of agreement of the error distribution for M, $J_{0}$, and $J_{45}$, respectively. The wavefront-aberrometry related variables had the biggest impact on the prediction, while demographic and measurement quality-related features showed a heterogeneous but consistent predictive value.

**Conclusions:**

These results suggest that ML is effective for improving precision in predicting patient's SR from objective measurements taken with a low-cost portable device.

## Introduction

According to the Vison Loss Expert Group (VLEG), it is expected that 1.7 billion people will be affected by moderate or severe vision impairment by 2050,^1^ the lead cause being uncorrected refractive errors (URE).^2^ URE is a reversible condition that carries significant individual and societal costs.^3^ For people suffering from URE, sight can be restored with refractive surgery, contact lenses, or with appropriate eyeglasses, the latter being one of the most cost-effective healthcare solutions.^4^ Despite the strong impetus to correct UREs and given their impact on achieving the United Nations (UN) Sustainable Development Goals (SDGs), they surprisingly remain historically underfunded relative to other diseases of the eye.^5^ Their prevalence remains high, particularly in low- and middle-income countries (LMICs), where vision exams can be highly inaccessible due to a shortage of eyecare professionals (ECPs) capable of providing clinical-quality eyeglass prescriptions.

The gold standard for prescribing eyeglasses is subjective, or manifest refraction. This is a time-consuming procedure that starts with an objective assessment of the refractive error by means of retinoscopy (in low-resource settings) or autorefraction (in high-resource settings), followed by a subjective refinement until the best corrected visual acuity is obtained. Accuracy of manifest refraction heavily relies on the experience of an ECP and the comprehension and visual perception of the subject. Repeatability of refractive error measurements in clinical settings has a high degree of variability that has been reported to range from 0.16 D to over 0.78 D in spherical equivalent (average differences).^6^^,^^7^

Wavefront aberrometry is an objective method for measuring refractive power. It provides a detailed map of the eye containing a description of both low- (LOA) and high-order (HOA) aberrations. The shape of the aberrated wavefront is commonly described using the Zernike polynomials, the standard method to represent the error in the wavefront of an optical system with circular pupil.^8^ Wavefront aberrometers have demonstrated high accuracy^9^ and higher repeatability than manifest refraction,^10^^,^^11^ but they continue to fail to match the gold standard. These discrepancies have been associated with several phenomena such as inter-ECP variability,^6^ influence of high-order aberrations,^12^^,^^13^ effects of iris color on the infrared light employed in wavefront aberrometry,^14^ accommodation during the measurement,^15^ or neural compensation of the refractive error.^16^^,^^17^

The relationship between the optical image on the retina and human visual perception continues to be a primary focus in vision research. Computational approaches attempting to predict subjective refraction from wavefront aberrometry data using retinal image quality metrics,^12^^,^^13^^,^^18^ or novel wavefront fitting methods,^19^ have proven to perform better than standard pupil plane metrics, yet continue to generally not be considered accurate enough to substitute the gold standard.^20^

During the past few years, ML algorithms have experienced an exponential boost in performance, accuracy, and community support.^21^ In the field of optometry, ML has been employed to predict subjective refraction using the Zernike coefficients with deep learning techniques^22^ or using extreme gradient boosting with a new series of polynomials for describing the wavefront map.^23^^,^^24^ These approaches, which are largely at research stage, have shown that significant improvements in accuracy can be achieved when using aberrometry data obtained by benchtop systems in highly controlled clinical and research environments. Unfortunately, such cost-prohibitive requirements fail to address the access barriers faced by healthcare disparities populations. Furthermore, refractive errors are also known to be correlated to demographic factors (particularly age),^25^ that can be interpreted by the models and potentially used to improve prediction power.

The primary aim of this work was to assess the performance of different machine learning ensemble models to predict patient subjective refraction using wavefront aberrometry and demographic variables. In contrast with the previous studies, data used for this work was obtained from a clinical study performed in 2015 at Aravind Eye Hospital in Madurai and a rural satellite vision center in Thiruppuvanam, India. The objective of that study was to evaluate the quality of eyeglass prescriptions provided by a low-cost portable wavefront autorefractor,^26^ an early prototype of the QuickSee (QS) (PlenOptika Inc, USA), operated by a minimally trained technician in a low-resource setting on a population with high age and refractive error variability. The secondary aim of this study was to understand if ML-based approaches, when applied to real-world data obtained with low-cost handheld ophthalmic devices could potentially increase access to accurate, clinical-quality SR prescriptions when operated by non-experts.

## Materials and methods

### Dataset description

A total of 708 subjects were enrolled in the study, 506 from the base hospital (HA) and 202 from the vision center (VA). Inclusion criteria were patients with ages ranging between 15 and 70 years and within the measurement range of the device used in the study (spherical equivalent of −6 D to +10 D). Exclusion criteria included presence of mature cataract, any prior eye surgery, any major eye illnesses, and use of systemic or ocular drugs which may affect vision. Subjective refraction results, QS measurements, and demographic data were recorded for all subjects eligible for the study. Clinical refraction procedure included streak retinoscopy and subjective refraction by an experienced refractionist. Further details about the study protocols can be found in Durr et al.^26^ Patients with missing data, like age or gender, or with the presence of cataract or other problem that prevented the autorefractor from providing a reliable prescription were removed from the dataset.

### Autorefractor

One distinctive feature of QS compared to standard desktop autorefractors is that it works in video mode. Instead of providing an eyeglass prescription from a single wavefront image (or the average of several static images), it records a large sequence of Shack-Hartmann spot patterns over a 10 s video and applies advanced algorithms to process the sequence.^27^ Consequently, this device can provide not only standard aberrometry results (Zernike coefficients), but also a set of measurement-related quality metrics, such as the standard deviations of pupil size and pupil center in X and Y directions during the acquisition, which were also used for training the different models. Additional information about the device working principle can be found here.^28^

### Machine learning models

Although in the original study 3 measurements for each eye were acquired, in this work only the first measurement of each eye was used to avoid training the ML algorithms with redundant data. To account for instances where there were significant differences between the 3 different measurements of each eye, one of the two additional measurements were included in the dataset. The threshold to include this additional data was a spherical equivalent difference of ≥ 0.5D between the three measurements (standard deviation ≥ 0.5D) of the same eye. Of the two additional measurements, the one with the biggest difference from the first measurement was selected. Consequently, each patient contributed a minimum of 2 measurements (1 per eye) and a maximum of 4 measurements (2 per eye).

Choosing the proper number of observations to be used for training and testing does not follow a general rule. In our case, due to the relatively small dataset, it was decided to use 70% of the subjects for training and the remaining 30% for testing. Partitioning was performed using stratified sampling to ensure similar distributions of age, gender, and spherical equivalent error between all sets. As an additional validation step, all measurements for both eyes for a given patient were placed either in training or testing to prevent information leakage.

The performance of the ML models is known to be very sensitive to the quality and number of features considered for predictions.^29^ Redundant or unrelated variables can reduce the accuracy of the models and increase its complexity causing overfitting. In this work, we only employed prediction variables known to influence subjective refraction Table 1. provides a description of the variables employed to train the models as well as the rationale behind the decision. Specifically, input features were separated into aberrometry data, measurement quality related metrics, and demographic data.

**Table 1:** Description and justification of the wavefront aberrometry, measurement quality metrics, and demographic variables used to train the models.

	Variable Name	Description	Justification
Wavefront			
Aberrometry	[$Z_{2}^{-2},\;\ldots ,\;Z_{5}^{5}$]	Zernike coefficients up to 5th order without the Piston, Tilt, and Tip since they are not used for computation of low- and high-order aberrations.	Zernike coefficients are the standard method to describe the optical aberrations of the eye.^8^
	[PS_Avg]	Average pupil size along the 10 s measurement.	Pupil size strongly influences magnitude of HOAs.^36^
Measurement quality metrics	[PS_Std]	Standard deviation of pupil size along the 10 s measurement with the QS prototype.	Changes in pupil size are usually related to accommodation.^37^
	[PC_Std_X, PC_Std_Y]	Standard deviation of the center of the pupil in X and Y directions during the measurement.	Misalignments between the eye and Shack-Hartman sensor will capture (and refract) the periphery of the pupil. Central and peripheral refractions can be different in some populations.^38^^,^^39^
Demographic	[Age]	Patient's age.	Prevalence of refractive errors^25^ and accommodation range^40^ are strongly correlated to age.
	[Gender]	Patient's gender.	Prevalence of refractive errors has been reported to have some degree of correlation with gender.^41^^,^^42^

Four ensemble models were trained and tested for each power vector component: random forest regressor^30^^,^^31^ (RF), gradient boosting regressor^30^, ^31^, ^32^ (GB), extreme gradient boosting regressor^33^ (XGB), and a custom assembly model (ASB) that averages the predictions of RF, GB and XGB. Ensemble regression techniques, like averaging the output of several models, are known to reduce the variance of the final prediction.^31^ Since selection of the best model is linked to the selection of the best features, we performed an extensive grid search; for each combination of input features, each model underwent hyperparameter tuning using a 5-fold randomized cross-validation using the mean absolute error as scoring function. The impact of individual features in the predictions of the models was estimated using permutation-based algorithms. Permutation values were also obtained using 5-fold cross-validation in the training set.

The research code was developed in JupyterLab (2.1.5) IDE using Python (3.8.3). Data preprocessing, aggregation, and cleaning in preparation for machine learning was implemented with Pandas (1.0.5) and Numpy (1.18.5). Machine learning models Random Forest and Gradient Boosting were from Scikit-learn (0.23.1) and Extreme Gradient Boosting from XGBoost (1.2.1).

### Statistical analysis

Statistical analysis was performed on the power vector domain using the predictions from ML models and the autorefractor on the test dataset. Subjective refraction prescriptions were converted to power vector parameters of spherical equivalent (M), vertical Jackson cross cylinder ($J_{0}$), and oblique Jackson cross cylinder ($J_{45}$) .^34^ In the original study,^25^ samples used for statistical analysis were the median of three measurements for the right eye. This differs from the analysis in this paper, in which we used individual measurements from both eyes for the calculations.

Three statistical procedures were followed to measure agreement between predictions and subjective refraction. First, Bland-Altman analysis was performed since it is the standard procedure when evaluating the accuracy and precision of two separate measurement methods (in this case, predicted refraction compared to subjective refraction).^35^ Second, overall prediction error was measured in terms of mean absolute error (MAE) and root mean squared error (RMSE), a metric more sensitive to outliers. Finally, we evaluated the percentage of agreement between predictions and subjective refraction for 0.25 D and 0.5 D thresholds.

## Results

### Population

**Fig. 1:**
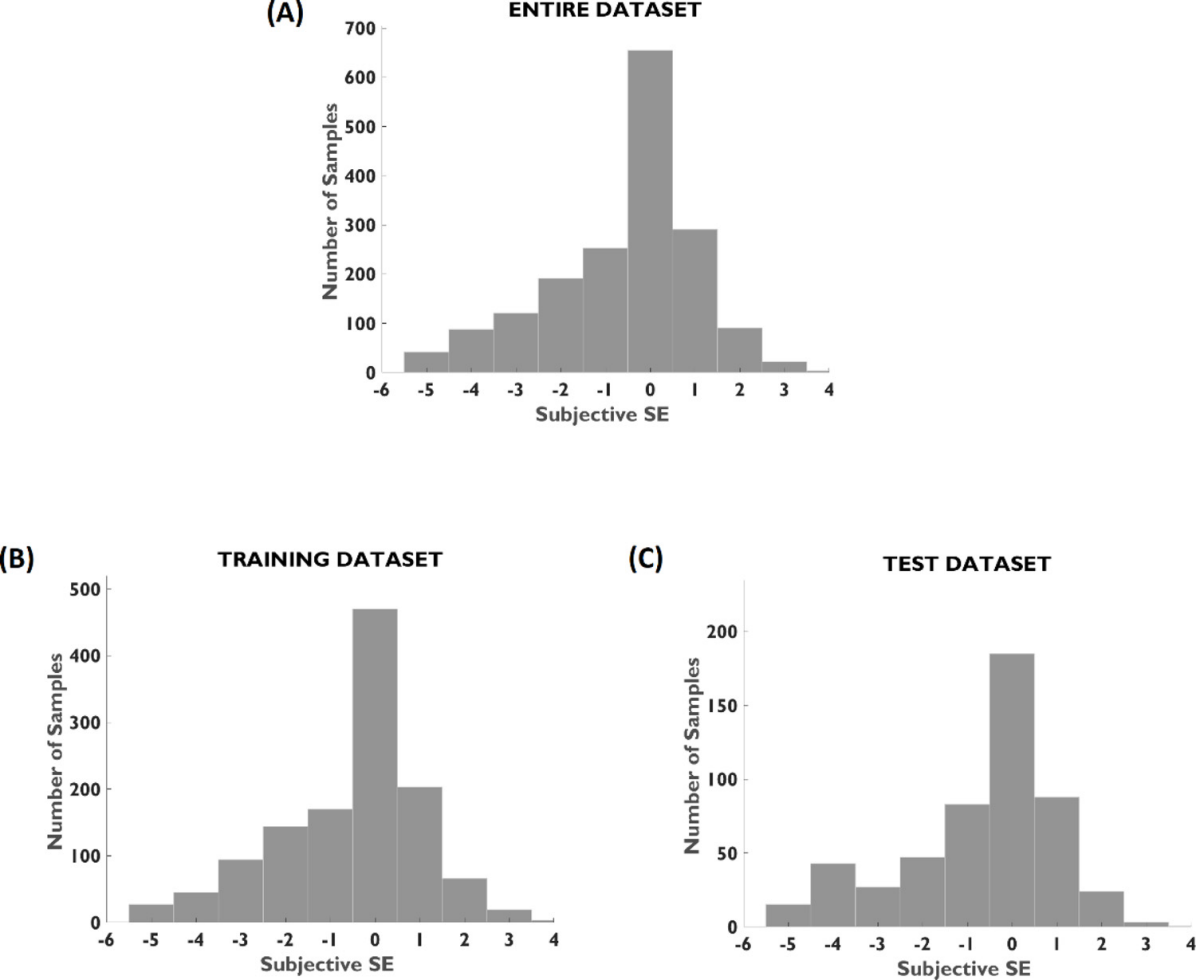
Distribution of spherical equivalent errors in patient population. (A) Entire dataset, (B) training dataset, and (C) testing dataset. Histograms bin size is 1.0 D. Refraction data was obtained by standard subjective procedure.

After data preprocessing and cleaning, 669 subjects (94.5% of overall patient dataset) remained for the analysis. A demographic description and distribution of spherical equivalent errors of the patient population is reported in Table 2 and Fig. 1, respectively. The proportion of age, gender, and spherical equivalent is comparable between training and testing groups.

**Table 2:** Population demographics characteristics and spherical equivalent error distribution.

Characteristics	Entire Dataset	Training Set	Testing Set	T test (*p*-value)Training vs Testing
Number of Subjects	669	468	201	–
Number of Samples	1762	1244	518	–
Female n (%)	391	269	122	0.31
	(58.4)	(57.5)	(60.7)	
Age ± SD (range)	35.2 ± 13.7	35.5 ± 13.6	34.5 ± 13.7	0.15
[years]	(15 - 70)	(15 - 70)	(16 - 64)	
SE ± SD (range)	−0.7 ± 1.67	−0.67 ± 1.62	−0.83 ± 1.77	
[diopters]	(−6.0 - 3.5)	(−5.75 - 3.5)	(−6.0 - 2.63)	0.06

### Agreement with subjective refraction

The four algorithms performed similarly, but the ASB model (formed by the mean of the predictions of RF, GB, and XGB) slightly reduced the error, while keeping the same percentage of agreement as the other three models. Therefore, the results discussed below will all refer to the ASB model. Detailed results for each model are shown in summary Table 3.

**Table 3:** Agreement comparison between ML models predictions and autorefractor with subjective refraction for M, $J_{0}$, and $J_{45}$. ASB model provided the best results for the three power vectors. *LOA: Limits of agreement calculated as 1.96 x Standard Deviation of the differences.

	Objective Dynamic + Demographic Features								
Model		**MAE**	**RMSE**	**Min.**	**Max.**	**Agreement (%)**		**Bland-Altman (D)**	
						**0.25 D**	**0.50 D**	**Mean**	**95% LOA**
QS	M	0.65	0.84	−3.19	2.33	25.87%	44.79%	−0.13	−1.75, +1.49
	$J_{0}$	0.17	0.24	−1.59	0.99	76.25%	96.71%	0.01	−0.47, +0.48
	$J_{45}$	0.12	0.17	−0.63	0.68	88.22%	98.45%	0.02	−0.31, +0.34
RF	M	0.39	0.54	−1.70	2.45	45.17%	72.01%	0.17	−0.83, +1.17
	$J_{0}$	0.13	0.17	−0.81	0.69	88.80%	97.88%	0.00	−0.34, +0.34
	$J_{45}$	0.07	0.13	−0.81	0.87	94.02%	99.23%	0.01	−0.24, +0.26
GB	M	0.40	0.54	−1.55	2.29	41.50%	69.31%	0.13	−0.88, 1.15
	$J_{0}$	0.13	0.17	−0.71	0.75	89.18%	98.26%	−0.01	−0.35, +0.32
	$J_{45}$	0.07	0.13	−0.88	0.99	94.40%	99.23%	0.00	−0.25, +0.25
XGB	M	0.40	0.53	−1.70	2.66	44.01%	71.62%	0.15	−0.86, +1.15
	$J_{0}$	0.12	0.17	−0.71	0.74	89.58%	98.06%	0.00	−0.34, +0.33
	$J_{45}$	0.07	0.14	−0.88	0.99	94.59%	98.46%	0.01	−0.26, +0.28
ASB	M	0.38	0.52	−1.55	2.40	45.56%	72.90%	0.15	−0.83, +1.13
	$J_{0}$	0.11	0.16	−0.74	0.72	89.10%	98.64%	−0.01	−0.34, +0.32
	$J_{45}$	0.07	0.13	−0.81	0.97	95.27%	99.23%	0.01	−0.24, +0.25

ASB model significantly improved the agreement with subjective refraction compared to the baseline autorefraction results. The mean absolute error (MAE) decreased by 42.5% (0.27 D), 29.4% (0.06 D), and 41.7% (0.05 D), for M, $J_{0}$, and $J_{45}$, respectively. The RMSE was also reduced in similar proportions (38.1% (0.32 D), 29.2% (0.08 D), and 23.5% (0.04 D), for M, $J_{0}$, and $J_{45}$, respectively. In terms of percentage of agreement between predictions and SR, the proportion of outliers (≥1 D) in the spherical equivalent predictions was reduced from 20.3% in the baseline model to 5.2% in the ASB model. Furthermore, the percentage of agreement for a 0.5 D threshold increased from 44.8% (QS-SR) to 72.9% (ML-SR) for spherical equivalent refraction.

**Fig. 2:**
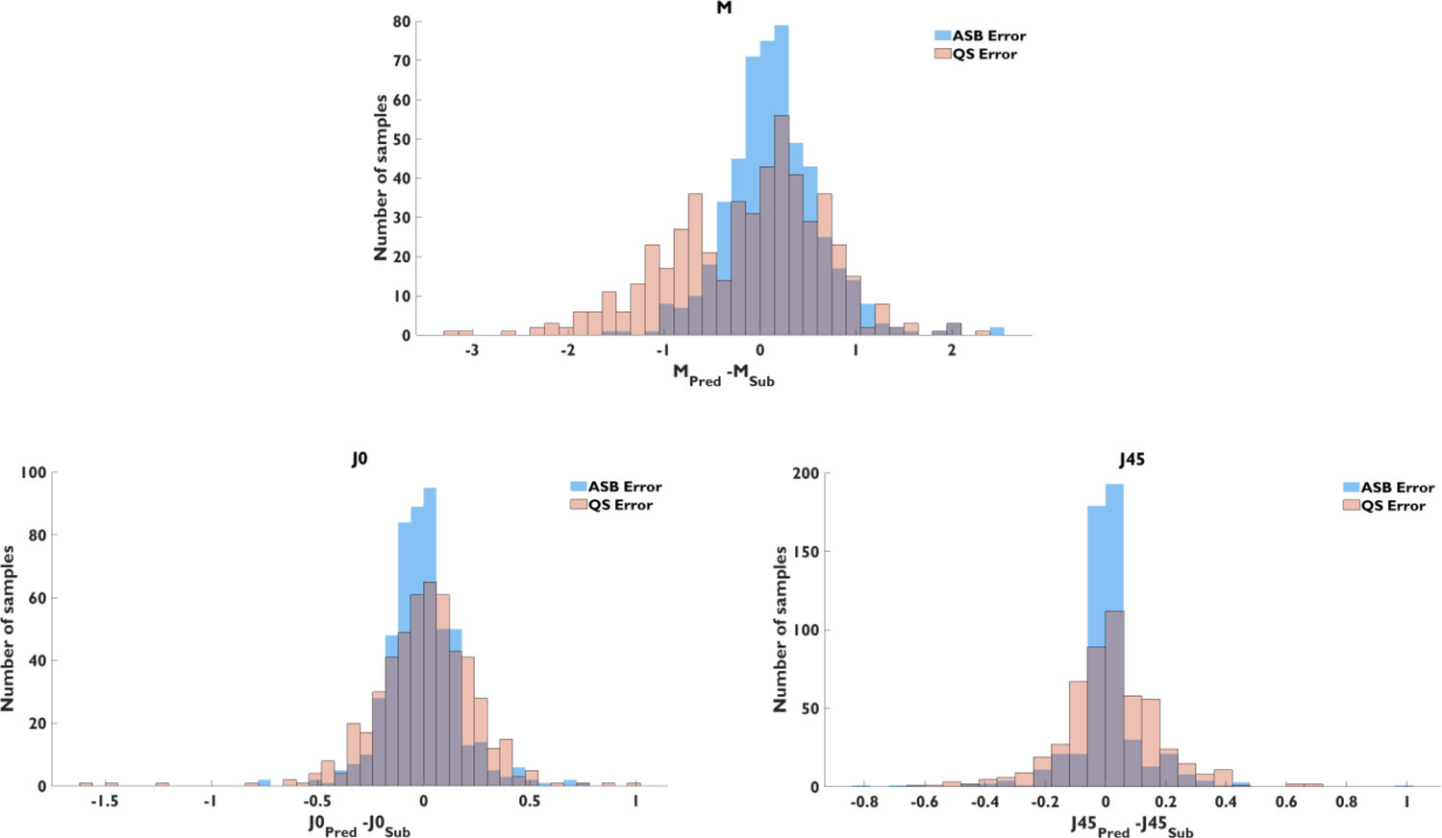
Distribution of the prediction error for M, $J_{0}$, and $J_{45}$ of the assembly model (blue) and autorefractor (orange).

Fig. 2 contains a histogram of the prediction error of ASB and QS models for the three power vectors. The plots show the effect in prediction accuracy and precision of the machine learning model. The general trend to underestimate the M value in the baseline model was mostly corrected by the ASB model, but the percentage of overestimation was similar in both cases.

**Fig. 3:**
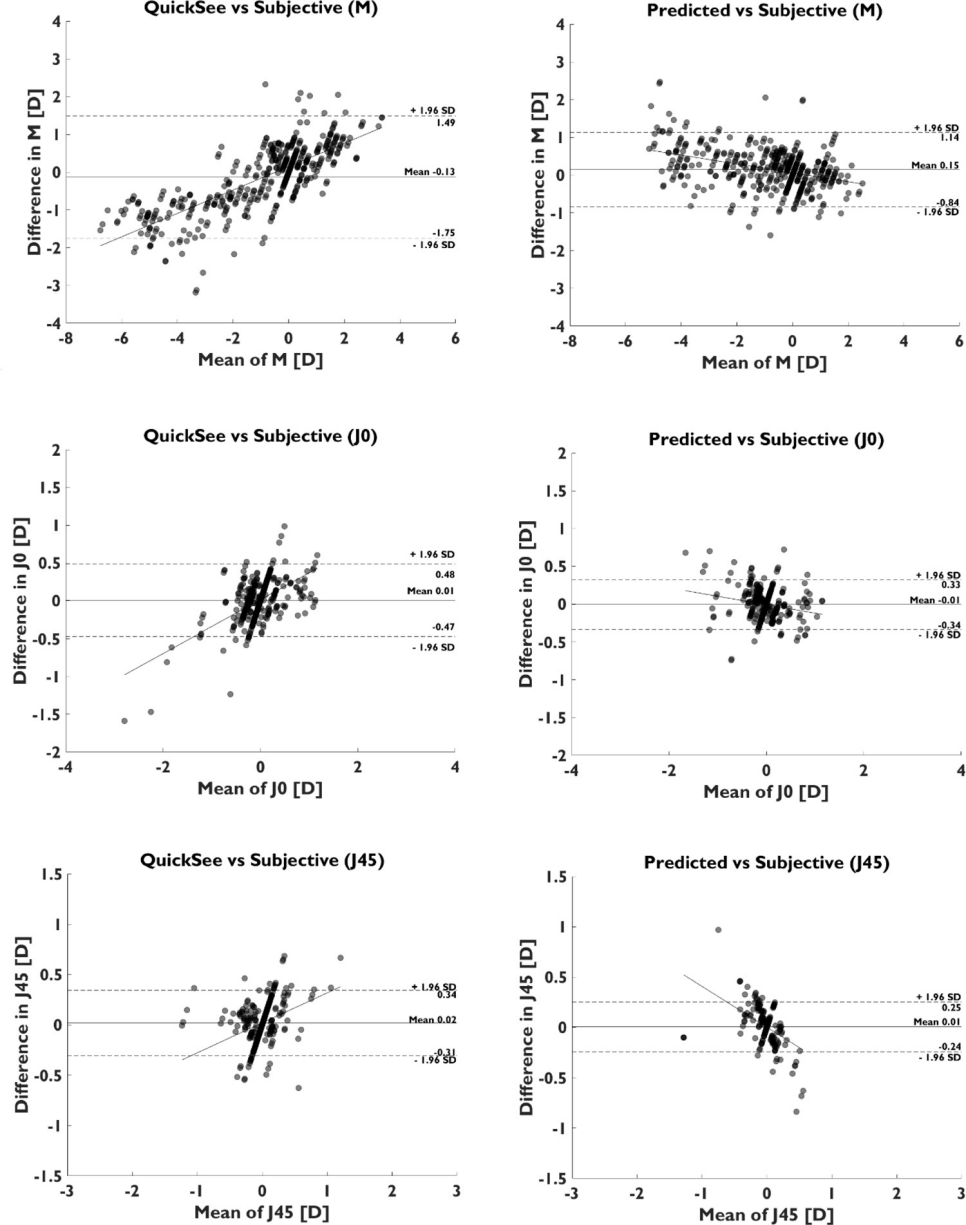
Bland-Altman plots comparing the agreement between prescriptions provided by the autorefractor (left) and ASB assembly model (right) versus subjective refraction for M, $J_{0}$, and $J_{45}$.

Bland-Altman analysis showed a bias between ASB and SR predictions of 0.15 D, −0.01 D, and 0.01 D, for M, $J_{0}$, and $J_{45}$, respectively (Fig. 3). The 95% limits of agreement (calculated as 1.96 x Standard Deviation of the differences) between ASB and SR were ±0.99 D, ±0.33 D, and ±0.25 D, for M, $J_{0}$, and $J_{45}$. Compared to the Bland-Altman diagram of the baseline model, the 95% limits of agreement of predictions were reduced by ±0.63 D, ±0.14 D, and ±0.08 D, for M, $J_{0}$, and $J_{45}$.

Fitting a linear regression model to each Bland-Altman (Fig. 3) provided a slope of −0.12, −0.11, and −0.40, for M, $J_{0}$, and $J_{45}$, for the assembly model, compared to a slope of 0.31, 0.35, and 0.30, for M, $J_{0}$, and $J_{45}$, for the autorefractor predictions. The change in slope sign indicates a trend for under correction of the ASB model in both myopic and hyperopic regions, and the flattening of the slope for the spherical equivalent indicates higher agreement with SR in the edges of both measurement ranges. Bland-Altman plots for the different power vector components (Fig. 3) clearly shows this change in the distribution of the differences and its effect in the 95% limits of agreement, which are considerably improved in all the cases.

### Feature importance

**Fig. 4:**
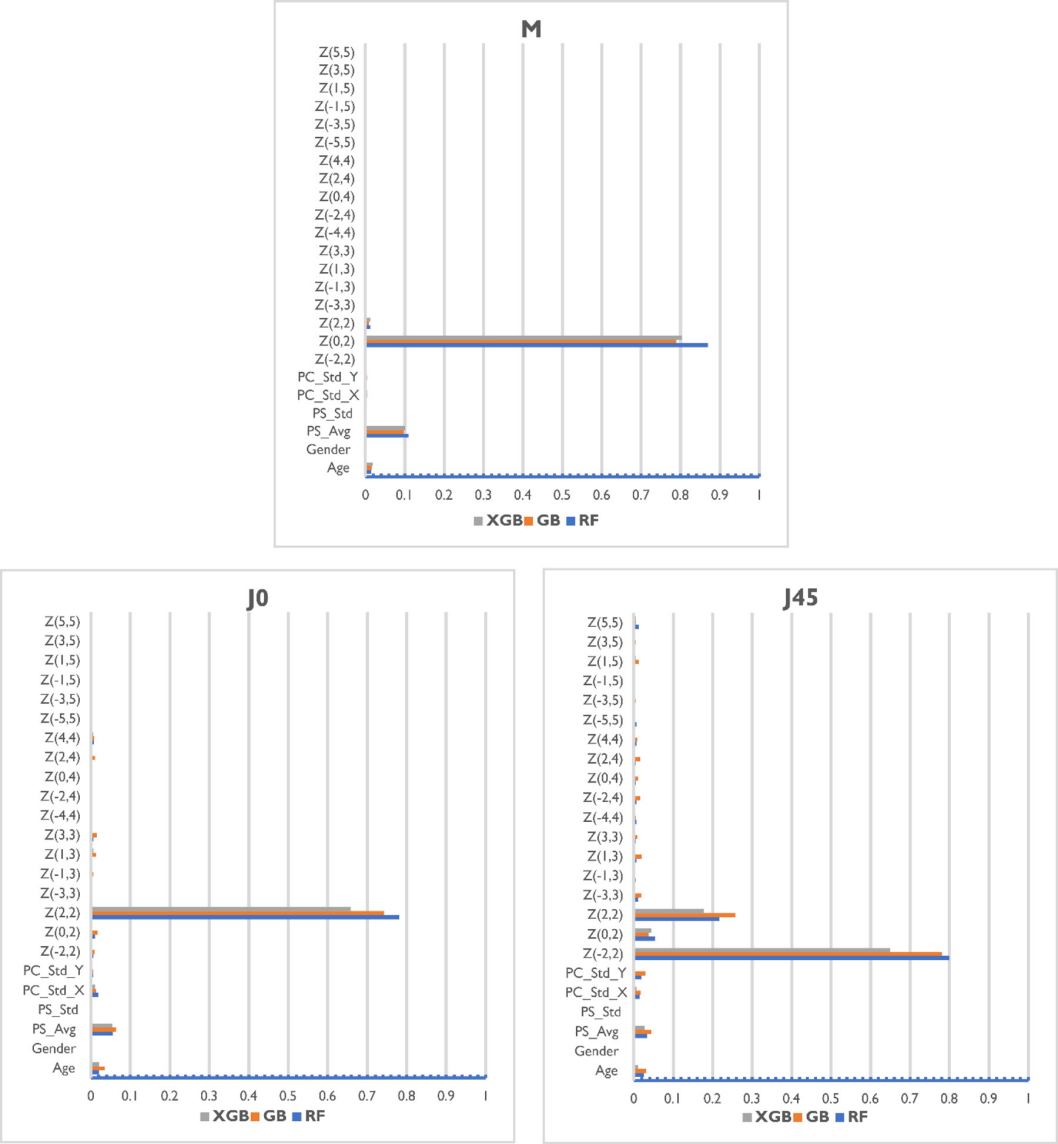
Permutation-based feature importance of Random Forest (RF), Gradient Boosting (GB), and Extreme Gradient Boosting (XGB) models for predicting the three power vectors M, $J_{0}$, and $J_{45}$. Permutation values were obtained using a 5-fold crossvalidation on the training set.

In general, for all the models evaluated the most relevant features for predicting individual power vectors were Zernike coefficients $Z_{2}^{0}$ (defocus), $Z_{2}^{2}$ (vertical astigmatism), average pupil size, and age for spherical equivalent; Zernike coefficient $Z_{2}^{2}$ (vertical astigmatism), average pupil size, and age for vertical Jackson cross cylinder ($J_{0}$); and Zernike coefficients $Z_{2}^{-2}$ (oblique astigmatism), $Z_{2}^{2}$ (vertical astigmatism), average pupil size, average deviation in pupil center and age for oblique Jackson cross cylinder ($J_{45}$) (Fig. 4).

The three base models provided different levels of importance to each feature but demonstrated agreement on the most relevant parameters. Regarding the demographic variables, the age was in general an important piece of information for all the power vector components being predicted, while gender was found to be practically irrelevant. Measurement quality metrics included were relevant in the prediction, especially the average pupil size, which was of interest for all the models, and the standard deviation of the pupil center was of interest for $J_{45}$ models.

## Conclusions

This study evaluated the prediction power of four different machine learning ensemble models to estimate manifest refraction from wavefront aberrometry data, demographic factors, and measurement-quality metrics. Agreement with subjective refraction for M, $J_{0}$, and $J_{45}$ was considerably improved compared to the baseline results provided by the autorefractor. Similarly, the variance of the predictions (95% limits of agreement) was reduced for the three power vector components while keeping the bias error close to zero. This improvement in accuracy is also reflected in the percent of patients in which the prediction is within 0.25 D and 0.50 D of subjective refraction (25.8% to 45.5% and 44.7% to 72.9% for 0.25 D and 0.50 D thresholds, respectively).

The most relevant feature for each of the models was the Zernike coefficient associated to the power vector the model was trying to predict: $Z_{2}^{0}$ for the M, $Z_{2}^{2}$ for $J_{0}$, and $Z_{2}^{-2}$ for $J_{45}$, as was expected. The second most important parameter considered by all models was the average pupil size during the measurement. This is not surprising considering that pupil diameter is a significant autorefraction feature used to estimate corrective lenses. The third most influential feature was the age, accounting for ∼5% of the weight of the prediction. Age is a crucial parameter in datasets containing young children due to factors such as childhood-hyperopia, which in general is very difficult to diagnose due to the high accommodative capacity of children. Specific high-order Zernike coefficients were not relevant for the models, but $Z_{2}^{2}$ was taken into account by the M and $J_{45}$ models. Gender was not considered a remarkable feature by any of the three models.

Decrease in the mean absolute error of the M, $J_{0}$, and $J_{45}$ predictions (0.27 D, 0.06 D, and 0.05 D) for the ASB model was bigger than those values obtained by Rampat et al.^23^ using aberrometry data (0.10 D, 0.05 D, and 0.05 D). However, it should be noted that this study was performed under highly controlled conditions using data from a desktop aberrometer, and a new base of polynomials that provide better separation between low- and high-degree wavefront components than standard Zernike polynomials. Apart from these differences, the original error of the baseline model in our study was higher, what could be explained by the heterogeneity in the population characteristics (wider inclusion criteria), and the fact that the instrument used for the study was an early prototype of the current production device operated by a minimally trained technician. Finally, many of the subjects enrolled in this study were emmetropic with 29.56% of the samples within 0.25 D of spherical equivalent. Having a population with a more heterogeneous refractive error distribution would have been beneficial for the models, but this naturally-occurring distribution has been found to resemble those obtained in other studies.^43^^,^^44^

Results shown in this paper show a considerable improvement in the agreement with SR when compared to the autorefractor predictions obtained by means of the paraxial matching method. An extra validation step would include a comparison of the ASB model against other SR prediction methodologies like image quality metrics^13^^,^^18^ or the MTR metric from Jaskulski et al.^19^ when executed on the same dataset.

Direct application of these models to patients implanted with multifocal intraocular lenses (IOLs) is not possible due to the limitations of Shack Hartman sensors to separate the overlapping wavefronts generated by the diffractive lenses. A different representation other than the Zernikes would be required, and the models would need extra training and tuning for each specific multifocal IOL.

The results obtained in this work suggest that a ML approach, implementable via software, may potentially improve upon the accuracy of the handheld autorefractor used in the study in a cost-effective manner. An important point to note is that since the most influential variables for the models were the Zernike coefficients, pupil size and age (standard variables in aberrometry), this approach could potentially be extended to other autorefractors, supporting the use of the proposed methodology to improve access to vision correction by non-technical ECPs in health disparity populations.

## Declaration of Competing Interest

E. Lage, S.R. Dave and D. Lim are inventors in patents related to the device presented in this work and have ownership in PlenOptika Inc. A. Gil, C.S. Hernandez, I. Casares and J. Poderoso work or have worked in research and development projects funded by PlenOptika Inc or are employees of this company.
